# Evaluation of the Crushing Characteristics of Slate Coarse Aggregate Used for Asphalt Mixture

**DOI:** 10.3390/ma19030503

**Published:** 2026-01-27

**Authors:** Hao Huang, Yanfei Zhu, Kun Zhou, Yue Xiao, Liantong Mo

**Affiliations:** 1 State Key Laboratory of Silicate Materials for Architectures, Wuhan University of Technology, Wuhan 430070, China; 344828@whut.edu.cn; 2 Gezhouba Group No. 1 Engineering Co., Ltd., Yichang 443002, China; zhuyanfei5719@cggc.cn (Y.Z.); zhoukun0039@cggc.cn (K.Z.); 3 School of Materials Science and Engineering, Chang’an University, Xi’an 710061, China; xiaoy@chd.edu.cn

**Keywords:** asphalt mixture, slate, crushing characteristics, crushing strength

## Abstract

The relict bedding and slaty cleavage structure in slate directly influences the crushing characteristics and strength properties of slate aggregates. When slate aggregates are used in asphalt concrete, it may have risks of insufficient resistance to crushing and uncertain long-term durability. In order to investigate the crushing behavior of slate coarse aggregates in asphalt mixtures, a comparative study was conducted using limestone and basalt aggregates as reference. Various tests were carried out including crushing value tests, single-particle compression crushing tests, Marshall compaction resistance tests, and gyratory compaction resistance tests. The crushing patterns, crushing strength, and gradation changes of slate aggregates after crushing were systematically examined. Based on the Weibull distribution function, the statistical distribution of single-particle crushing strength was analyzed. Additionally, the particle distribution patterns were studied for single-sized aggregates, blended aggregates, and asphalt mixtures after these were subjected to crushing under Marshall compaction and gyratory compaction. The test results indicated that the crushing value of slate coarse aggregates was 9.2%, which indicates superior crushing resistance compared to traditional limestone and basalt. After long-term exposure to water immersion at 60 °C, high-pressure steam treatment, and heating at 220 °C, the increase in crushing value of slate coarse aggregates was less than 1.5%, indicating excellent water and heat resistance. The two-point and four-point crushing strengths of single particles of slate coarse aggregates were higher than those of limestone and basalt coarse aggregates, and the single-particle compression crushing strength followed the Weibull distribution pattern. Both single-sized and blended slate aggregates exhibited lower proportions of crushing during Marshall and gyratory compaction compared to basalt and limestone aggregates. Asphalt mixtures prepared with slate coarse aggregates also demonstrated better crushing resistance than those made with basalt and limestone, confirming that the bedding structure of slate does not cause excessive crushing in asphalt mixture. The obtained findings were limited to the tested slate aggregates from a single quarry and thus necessary performance verification should be conducted on slate aggregates from other sources before practical engineering applications.

## 1. Introduction

The construction of asphalt concrete pavements for highways requires a large amount of high-quality aggregates such as limestone, diabase, and basalt. However, some regions lack these premium aggregate resources but have abundant slate deposits. Utilizing locally available slate aggregates can help reduce project costs and accelerate the construction progress. Compared to other types of rock, slate exhibits cleavage planes, giving it pronounced anisotropy. Under external loads, it tends to split along these planes into thin sheets or plate-like structures [1,2]. Due to its splitting property, slate is primarily used in traditional applications such as roofing, walling slabs, and paving stones [3]. However, research on processing slate into aggregates for engineering applications remains limited. The main reasons are that slate’s relict bedding structure directly affects its crushing characteristics and strength properties. Additionally, slate is prone to weathering, posing risks such as insufficient resistance to crushing, high flakiness content, and uncertain long-term durability when used as road aggregates. Nevertheless, if properly treated, slate has potential applications as an aggregate substitute in concrete, base or sub-base material in road construction [4]. For instance, the Qinzhou region of Guangxi, China has abundant slate deposits with good rock integrity, large block sizes, and poor splitting properties, making them unsuitable for traditional slate building materials. Instead, they are mainly processed into aggregates for road bases and low-grade cement concrete. Currently, due to concerns over the insufficient crushing resistance and weathering resistance combined with the dynamic loads from construction compaction and repeated tire loads during pavement service, there is a risk of excessive aggregate crushing if the slate’s crushing strength is inadequate. Therefore, it is necessary to study the crushing resistance characteristics of slate aggregates used for asphalt concrete.

Currently, researchers have conducted a series of tests, including Brazilian splitting, uniaxial compression, and triaxial compression tests, to evaluate the mechanical properties of slate rock [5,6,7,8]. Numerous studies on the mechanics of slate rock have confirmed that its internal bedding structure significantly influences its strength and failure modes including shear failure deviating toward the bedding plane direction, slip failure along the bedding plane, and splitting failure along the bedding plane [9,10,11,12]. Additionally, slate faces issues related to water stability and weathering [13,14,15]. Water intrusion triggers physicochemical reactions that alter the mineral microstructure and composition [16,17,18]. Freeze–thaw cycles and water–rock interaction can lead to strength degradation in slate [19,20]. Slate’s bedding structure has multiple adverse effects on its strength, along with issues such as strength degradation due to water immersion softening and weathering. However, there remain considerable doubts about whether slate aggregates, processed by crushing massive slate into smaller particles, suffer from insufficient crush resistance and weathering durability. As the fundamental building block of the skeletal structure in asphalt mixtures, the crush resistance of aggregate particles is a critical mechanical property. It is required to exhibit excellent compressive crushing resistance during laboratory preparation, construction compaction, and throughout the pavement’s service life, thereby preventing particle breakage from compromising the mechanical performance of asphalt concrete. Consequently, the quality of crush resistance directly determines whether slate aggregates can be utilized in asphalt concrete pavements. Given the distinctive relict bedding structure of slate, the investigation of the crushing characteristics and strength properties of slate aggregates is particularly essential for their application in asphalt pavements.

Currently, the mechanical properties of slate rock have been well studied, while there are few studies on the mechanical properties of slate aggregate. It should be noted that slate rock and slate aggregate are in different scale of size and thus can have distinctly different mechanical properties with respect to crushing resistance. This is especially true when the effects of the inherent bedding structure can be significant on the strength of slate rock, but not for slate aggregate. At present, there is no research on whether the inherent bedding structure of slate rock affects the mechanical properties of slate aggregate particles after the processing and crushing of slate rock into aggregate particles. Furthermore, the resistance to fragmentation in the complex stress environment of asphalt mixtures remains unknown. This knowledge gap makes it difficult to comprehensively and accurately evaluate the engineering suitability of slate aggregates based solely on the crushing behavior of slate rock. To address this research gap, this study aims to systematically evaluate the crushing characteristics of a specific slate coarse aggregate for use in asphalt mixtures. For the purpose of comparison, limestone and basalt aggregates commonly accepted and used in asphalt pavements were taken as references. Tests including the crushing value test, single-particle compression crushing test, Marshall compaction test, and gyratory compaction test were conducted on different aggregates to systematically study the crushing modes, crushing strength, and gradation changes of slate aggregate after crushing. The Weibull distribution function was employed to statistically analyze the distribution pattern of single-particle crushing strength. Additionally, the particle distribution patterns after crushing were analyzed for single-size aggregate, mixed aggregate, and asphalt mixtures subjected to Marshall compaction and gyratory compaction. Finally, by comparing with traditional limestone and basalt aggregates, this study verifies whether the relict bedding and slaty cleavage structure of slate would lead to excessive crushing and damage of the aggregate in asphalt mixtures, providing data support for the application of slate aggregate in asphalt mixtures.

## 2. Materials and Methods

### 2.1. Materials

**Figure 1:**
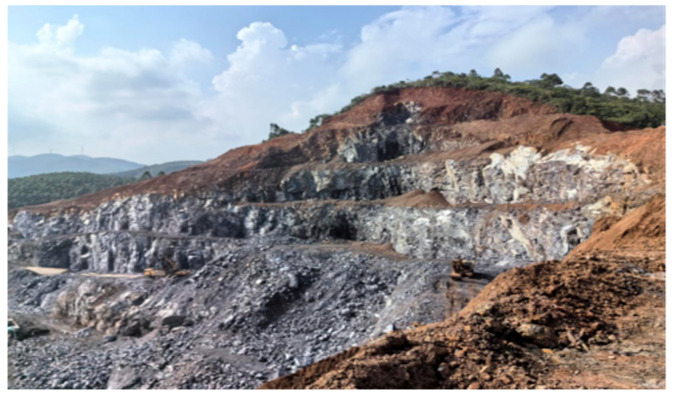
The on-site mining diagram of the slate vein.

**Figure 2:**
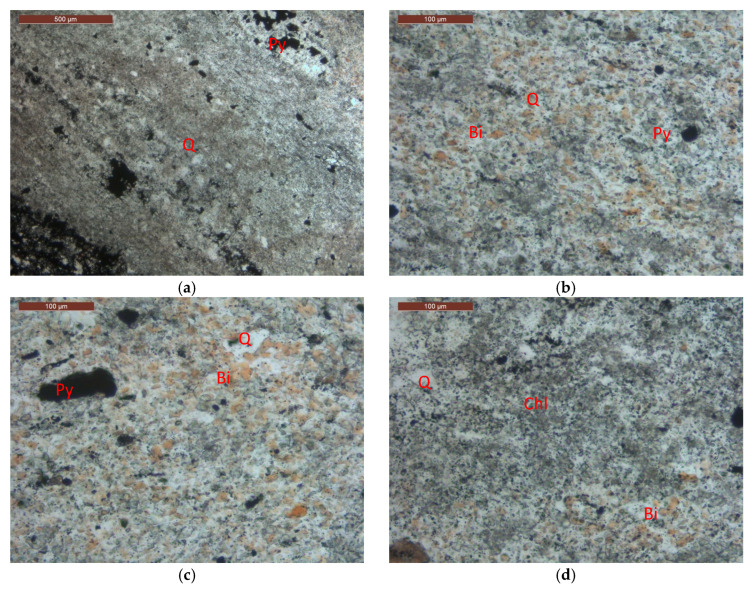
Petrographic analysis micrographs (**a**) Quartz (Q), opaque minerals (Py), and the banded structure of the rock; (**b**) Quartz (Q), biotite (Bi), and opaque minerals (Py); (**c**) Quartz (Q), biotite (Bi), and opaque minerals (Py); (**d**) Quartz (Q), chlorite (Chl), and biotite (Bi).

The slate coarse aggregate used was obtained from the De’an Quarry in Qinzhou, China. Figure 1 presents the on-site mining diagram of the slate vein. The slate primarily formed from the Mesoproterozoic to the Lower Paleozoic. Its metamorphism mainly occurred during the Sibaoian and Caledonian periods, resulting from low-pressure, regional, low-temperature dynamic metamorphism. The slate processed for the aggregate belongs to low-grade metamorphic rock, with its parent rock appearing gray-black. Petrographic analysis as indicated in Figure 2 showed that the mineral composition of the slate mainly included quartz (55%), biotite (21%), chlorite (16%), pyrite (8%), and opaque minerals. X-ray fluorescence spectrometry determined that the primary chemical components of the slate were SiO_2_ (53.36%), CaO (6.79%), Al_2_O_3_ (19.88%), Fe_2_O_3_ (5.92%), MgO (2.49%), K_2_O (6.45%), and Na_2_O (0.83%). The slate used in this study exhibited a characteristic cryptocrystalline texture and a dense massive structure. Its internal fabric was dominated by relict bedding inherited from the protolith, rather than a slaty cleavage formed by tectonic stress. In terms of mineral composition, quartz, accounting for up to 55% of the content, formed the rigid framework of the rock as cryptocrystalline grains, establishing a matrix of exceptionally high mechanical strength. During microstructural evolution, extremely fine cryptocrystalline minerals filled inter-granular spaces through recrystallization, enhancing the overall integrity of the matrix. More critically, these randomly distributed rigid grains effectively impeded the extensive, oriented, and continuous alignment of platy minerals (e.g., biotite, chlorite), thereby suppressing the development of a penetrative slaty cleavage at the microscopic scale. Although banded relict bedding formed by compositional differentiation was observable under the microscope, the high-strength cementation provided by the cryptocrystalline matrix prevented these bedding planes from evolving into mechanically weak parting surfaces.

**Figure 3:**
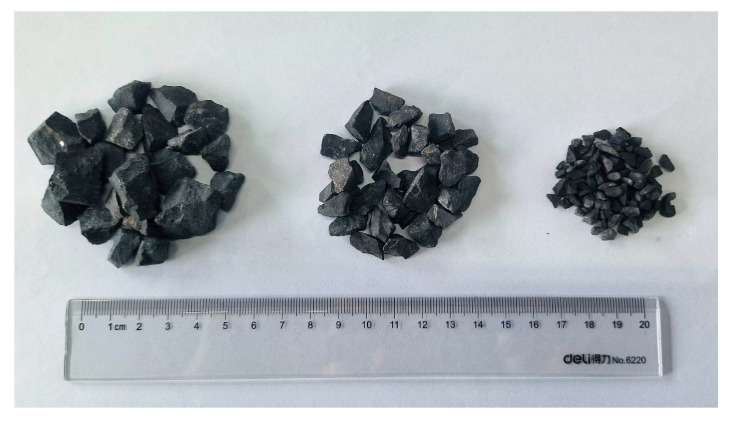
The coarse slate aggregates with different sizes.

As shown in Figure 3, the slate coarse aggregate consisted of three fractions: 2.36–4.75 mm, 4.75–9.5 mm, and 9.5–16 mm. For comparative analysis, high-quality limestone and basalt coarse aggregates commonly used in asphalt mixtures were selected for experimental study. Basalt is considered the high-quality aggregate for asphalt concrete, offering excellent crushing and wear resistance, while limestone is the most representative alkaline aggregate, with moderate crushing resistance. Furthermore, to compare the compressive crushing resistance of asphalt mixtures containing different rock types, asphalt mixtures were prepared using slate, limestone, and basalt coarse aggregates, respectively. In all three asphalt mixtures, limestone fine aggregate (0–2.36 mm) was used, the filler was mineral powder produced by grinding limestone, and the asphalt binder was penetration 70 paving asphalt with a penetration of 68 (0.1 mm), a softening point of 47.5 °C, and a ductility at 10 °C of 47 cm.

### 2.2. Test Methods

**Figure 4:**
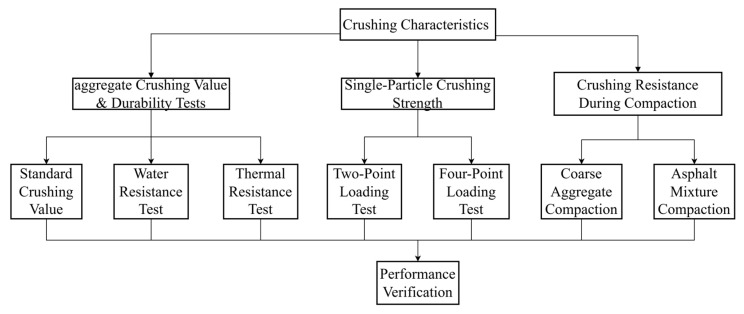
Research methodology flowchart.

Regarding the study of aggregate crushing characteristics, the aggregate crushing value is commonly used to characterize aggregates in asphalt mixtures [21]. Furthermore, the compaction methods such as Marshall compaction, gyratory compaction, and wheel rolling can also be used to examine the crush resistance of aggregates during the compaction process [22]. The mechanical properties of coarse granular materials are largely constrained by the compressive strength of single particles [23,24]. Therefore, the single-particle crushing strength test is an effective method for analyzing the crushing patterns and strength of aggregates. Figure 4 shows the research methodology flowchart. As shown in Figure 4, different crushing tests were considered including the crushing value test, single-particle compression crushing test, Marshall compaction test, and gyratory compaction test. Different material scales were also involved including single particle, single-grade aggregate, mixed aggregates and asphalt mixture. The test plan was aimed at getting insight into the resistance to fragmentation in the complex stress environment of asphalt mixtures ranging from single-particle loading to multi-scale aggregate skeleton compaction.

#### 2.2.1. Coarse Aggregate Crushing Test

To analyze the compressive resistance of slate aggregate and evaluate its suitability for asphalt mixtures, a crushing value test was conducted on coarse aggregates of three rock types including slate, limestone, and basalt in accordance with T0316-2024 Test Procedures for Highway Engineering Asphalt Aggregates [25]. The test utilized single-sized aggregates ranging from 9.5 to 13.2 mm. After applying a 400 kN load, the proportion of crushed material smaller than 2.36 mm was defined as the coarse aggregate crushing value. Since the crushing value alone is a single indicator and can not fully capture the crushing characteristics of different aggregates, the crushed material obtained from the test was further sieved to analyze its particle size composition after crushing.

The unique bedding structure and metamorphic characteristics of slate determine the risk of weathering and alteration of slate aggregate during long-term service. Therefore, tests were conducted to evaluate the water resistance and heat resistance of slate aggregate. The degradation of its compressive crushing resistance after long-term (21 days) immersion in water at high temperature (60 °C) was characterized using the coarse aggregate crushing value test. Meanwhile, an accelerated high-temperature and high-pressure test was performed to further examine its long-term water resistance. The slate aggregate was subjected to high-temperature autoclaving (autoclave steam pressure: 0.14–0.16 MPa, temperature: 126–128 °C, duration: 9 h), after which the change in the coarse aggregate crushing value was measured. The above-mentioned long-term immersion and high-temperature autoclaving tests were primarily designed to simulate whether slate aggregate would experience weathering and strength deterioration under long-term water immersion conditions during service in asphalt pavements.

During the production of asphalt mixtures, aggregates were heated to 170~190 °C in the drying drum of an asphalt mixing plant. To ensure the stability of the mechanical properties of aggregates after high-temperature heating, a high-temperature crushing value test was conducted on slate coarse aggregate. For the test, the slate coarse aggregate was first placed in the crushing value test mold, then put into an oven at 220 °C and kept at that temperature for 4 h. After removal, the loading test was carried out immediately as required, i.e., loading to 400 kN within 10 min to determine its crushing value. The slate aggregate, after being heated to 220 °C and held for 4 h, was directly subjected to the high-temperature compressive crushing test. This was intended to simulate the impact of high-temperature mixing, paving, and compaction of asphalt mixtures on the thermal stability of the slate aggregate.

The crushing value tests were conducted in triplicate, with the reported result being the average value of the three replicate tests.

#### 2.2.2. Single Particle Compression Crushing Test

**Figure 5:**
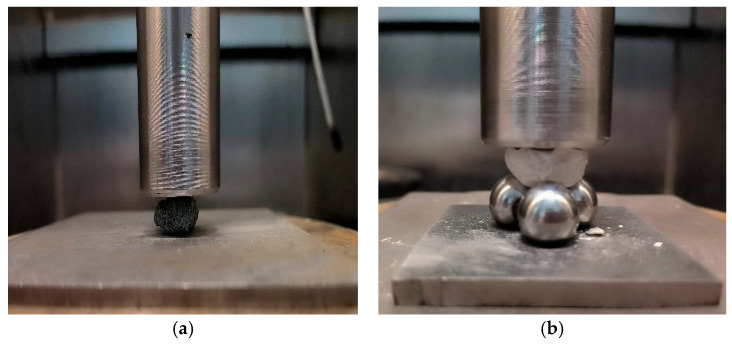
Single-particle compression crushing test ((**a**): two-point test; (**b**): four-point test).

**Figure 6:**
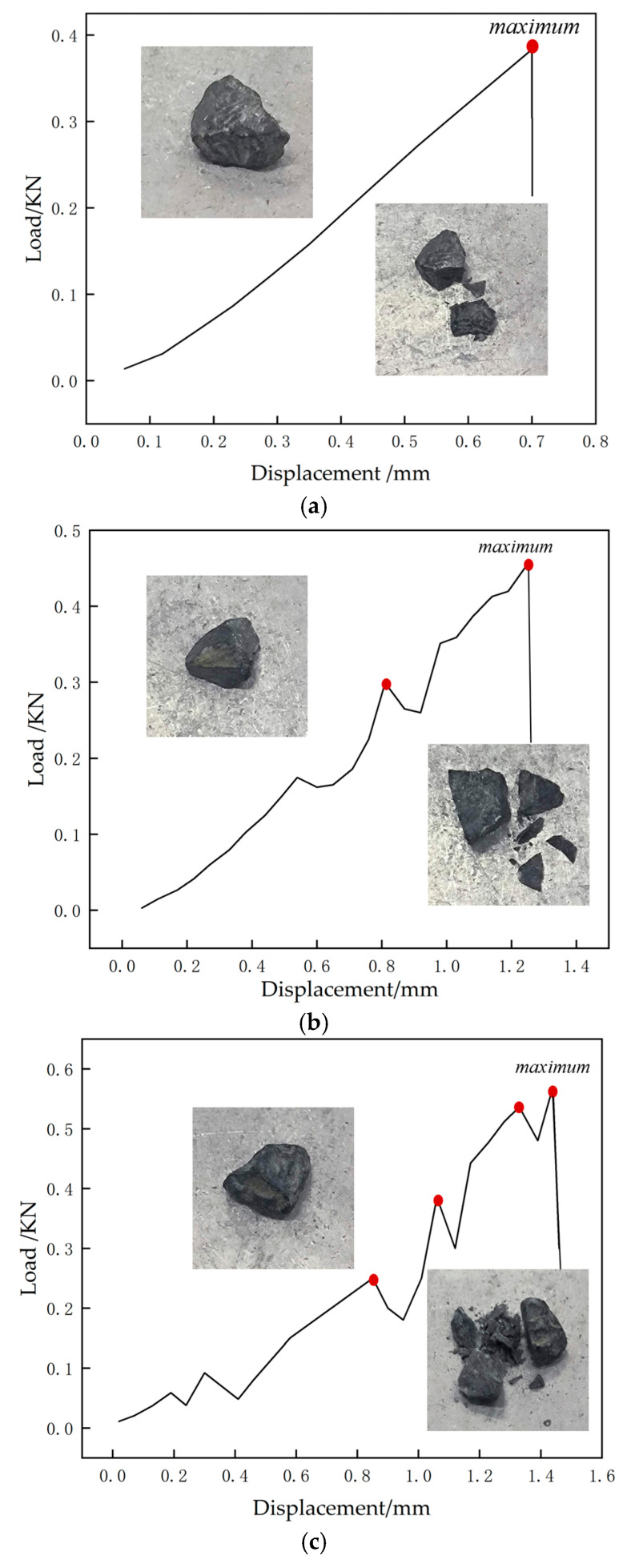
Typical load-displacement curves of slate single-particle compression crushing ((**a**): Single-peak curve; (**b**): Double-peak curve; (**c**): Multi-peak curve; red dots: maximum).

To analyze the single-particle compressive crushing strength of different coarse aggregates, single-particle two-point and four-point compression crushing tests were conducted. The loading methods are illustrated in Figure 5. In the single-particle two-point compression crushing test, an individual aggregate particle was placed between the base and the loading head, equivalent to the particle being crushed under two-point force. In contrast, the single-particle four-point compression crushing test employed three tangent steel balls arranged as a base, with the individual aggregate placed on the steel ball base to form three supporting points. The loading head applied pressure to create a four-point force mode. The size of the steel balls matched the particle size of the aggregates. For example, when conducting single-particle crushing tests for particles in the 4.75~9.5 mm range, steel balls with a diameter of 7 mm were used as the base. For particles in the 9.5~13.2 mm range, steel balls with a diameter of 12 mm were used. Compared to the single-particle two-point compression crushing test, the four-point compression crushing test involves more contact points and more complex force distribution on the aggregate, making it more effective for examining the influence of the inherent bedding structure of slate on the aggregate crushing strength. For the tests, 50 particles each of slate, limestone, and basalt in two size ranges including 4.75~9.5 mm and 9.5~13.2 mm, were selected. Since particle shape significantly affects crushing characteristics, particles with regular shapes and non-flaky, non-elongated forms (with a length-to-diameter ratio less than 1.3:1) were chosen for the compressive crushing strength tests. Before testing, the largest surface of each particle was placed in contact with the base. Loading was performed at a rate of 1 mm/min and stopped when the load dropped sharply to half of the maximum load. Some typical load-displacement curves are presented in Figure 6. During the loading process, the instrument automatically recorded the load and displacement and plotted the load-displacement curve.

The single-particle two-point compressive crushing strength can be calculated using the particle crushing strength formula proposed by Jaeger [26]:

(1) $$\sigma_{f}=\frac{F}{d^{2}}$$

where *F* is the crushing load force, and *d* is the particle diameter.

However, particles of the same size can have varying shapes, ranging from flaky to blocky. Simply using the average particle diameter to calculate the crushing strength may introduce bias. To analyze the influence of particle shape, this study employed a modified formula proposed by Afshar et al. [27], which incorporates the particle aspect ratio parameter to calculate the crushing strength of aggregate particles:

(2) $$\sigma_{f}=\frac{F\times AR}{d^{2}}$$

where *AR* is the particle aspect ratio.

For the single-particle four-point compressive crushing test, the compressive crushing strength is simplified using Equation (3) [28]:

(3) $$\sigma_{f}=\frac{F}{\pi (\frac{d_{\mathrm{int}}}{2})(\frac{d_{\min}}{2})}$$

where *d*_int_ and *d*_min_ are the intermediate diameter and minimum diameter of the aggregate particle, respectively.

#### 2.2.3. Coarse Aggregate Compaction Crushing Test

Currently, Marshall compaction tests and gyratory compaction tests are primarily used for laboratory compaction of asphalt mixtures. Both Marshall compaction and gyratory compaction can cause crushing damage to aggregates, thereby affecting the gradation and mechanical properties of asphalt concrete. To evaluate the crushing resistance of different coarse aggregates during the compaction process, Marshall compaction tests and gyratory compaction tests were conducted using single-sized and combined-sized coarse aggregates.

For the Marshall compaction test with single-sized coarse aggregates, around 800 g of aggregates with a particle size of 9.5–13.2 mm were selected. After washing, the aggregates were dried in an oven at 105 °C for 4 h, cooled to room temperature, and then placed into a Marshall mold. A Marshall electric compactor (Shanghai Changji Geological Instrument Co., Ltd., Shanghai, China) was used to apply 75 blows on one side. After compaction, the aggregates were removed and sieved to analyze particle breakage.

For the Marshall compaction test with combined-sized coarse aggregates, three fractions of coarse aggregates including 2.36–4.75 mm, 4.75–9.5 mm, and 9.5–16 mm, were used. Their proportions corresponded to the coarse aggregate gradation in AC-13 asphalt mixtures, representing a mineral aggregate composition without fine aggregates and mineral filler, consisting only of coarse aggregates larger than 2.36 mm to form an aggregate skeletal structure. The selected proportions for the 9.5–16 mm, 4.75–9.5 mm, and 2.36–4.75 mm coarse aggregates were 36.1:43.1:20.8. The passing percentages of the mixed aggregates through sieve sizes of 13.2 mm, 9.5 mm, 4.75 mm, and 2.36 mm were 91.7%, 63.9%, 20.8%, and 0%, respectively. After uniformly mixing the three coarse aggregates in the specified proportions, they were placed into a Marshall mold. Following 75 blows on one side, the crushed aggregates were removed and sieved to compare and analyze the changes in gradation before and after the compaction test.

The gyratory compaction test for coarse aggregate crushing resistance was similar to the Marshall compaction test. For the single-sized gyratory compaction test, aggregates with a particle size of 9.5–13.2 mm were washed, dried, and cooled, then carefully placed into a steel mold with an inner diameter of 150 mm in three layers, with the filling height controlled at approximately 115 mm. The gyratory compaction test was conducted with a compaction pressure of 600 kPa, a rotation speed of 30 r/min, and a constant rotation angle of 1.25 degrees. To simulate the effects of heavy traffic (equivalent to the maximum number of gyrations (N_max_) of 205 corresponding to a design traffic load of more than 30 million equivalent single axle loads (ESALs)), the number of gyrations was set to 205. After 205 gyrations, the test aggregates were removed from the mold and sieved to analyze the particle composition after crushing.

For the gyratory compaction test with combined-sized aggregates, the aforementioned mixed aggregates of 2.36–4.75 mm, 4.75–9.5 mm, and 9.5–16 mm were used. After 205 gyratory cycles, the crushed aggregates were removed and sieved to examine the changes in gradation before and after gyratory compaction.

#### 2.2.4. Asphalt Mixture Compaction Crushing Test

The aforementioned tests are conducted solely on aggregates to evaluate their crushing resistance, which differs somewhat from the actual preparation and formation of asphalt mixtures. Therefore, asphalt mixtures were prepared using coarse aggregates of slate, limestone, and basalt, respectively. During the preparation process, in addition to the coarse aggregates, fine aggregates of 0–2.36 mm were uniformly made of limestone fine aggregate; the filler was limestone mineral powder, the asphalt binder was penetration 70 paving asphalt, and the asphalt-to-aggregate ratio was 4.7%. All three asphalt mixtures prepared from the different rock types used the same mineral gradation of AC-13 asphalt mixture as listed in Table 1. To minimize gradation deviations, manually and strictly sieved single-sized coarse and fine aggregates were used for batching. To compare and analyze the potential crushing of the skeletal structures formed by different coarse aggregates during the compaction process, Marshall compaction tests and gyratory compaction tests were conducted on the asphalt mixtures. During the mixing of the AC-13 asphalt mixture, the aggregate heating temperature was 175 °C, and the asphalt heating temperature was 160 °C. The mixing temperature for the asphalt mixture was 160 °C, and the specimen compaction temperature ranged from 150–155 °C. When using the Marshall method for compaction, a Marshall electric compactor was used to apply 75 blows on each side. After compaction, the specimen was demolded while hot and the asphalt mixture was separated and placed in a combustion furnace to burn off the asphalt binder. After combustion, it was cooled to room temperature and sieved to examine the aggregate gradation. When using the gyratory compaction method, a gyratory compactor was used for 205 compaction cycles. After gyratory compaction, the specimen was similarly demolded while hot and the asphalt mixture was separated and placed in a combustion furnace to burn off the asphalt. After combustion, it was cooled to room temperature and sieved to examine the aggregate gradation. By comparing the changes in the aggregate gradation of the asphalt mixtures before and after compaction using the two methods, the crushing behavior of the different rock coarse aggregates was analyzed.

The Marshall compaction test and gyratory compaction test were conducted in triplicate, with the reported result being the average value of the three replicate tests.

## 3. Results

### 3.1. Coarse Aggregate Crushing Value

Table 2 and Table 3 present the crushing value test results and the particle size distribution after crushing for the three types of rock coarse aggregates, respectively. The test results showed that the crushing value for slate was 9.2%, for basalt 11.4%, and for limestone 19.8%. It should be noted that basalt is considered the high-quality aggregate that has excellent crushing and wear resistance, while limestone is the most representative alkaline aggregate with moderate crushing resistance. Overall, slate had the lowest crushing value, outperforming traditional limestone and basalt, indicating that slate coarse aggregate possessed excellent resistance to crushing. After 21 days of immersion in water at 60 °C, the crushing value of slate was 10.2%; after high-temperature autoclaving, it was 10.6%; and after heating at 220 °C for 4 h, it was also 10.6%. Compared to the conventional crushing value, the effects of high-temperature water immersion, high-temperature autoclaving, and high-temperature heating on the crushing value of slate were minimal. Reference [22] reported that the crushing values of basalt, steel slag, limestone, recycled aggregate, and marble aggregate were 9.6%, 16.8%, 20.2%, 22.6%, and 26.7% respectively. Compared with these aggregates, the slate used in this study exhibits a crushing value comparable to that of basalt and superior to those of the other aggregates.

The crushing value test used single-sized aggregates of 9.5–13.2 mm, while the aggregates showed significant crushing after the test. As shown in Table 3, the crushing characteristics of the coarse aggregates were significantly related to their rock type. For example, the particle size distribution of limestone in the 9.5–13.2 mm range was 22.9%, indicating that nearly 77% of the 9.5–13.2 mm aggregate particles were crushed into smaller aggregates. In contrast, the distributions for basalt and slate in the 9.5–13.2 mm range were 46.4% and 44.8%, respectively, indicating that approximately 55% of the 9.5–13.2 mm aggregate particles were crushed into smaller aggregates. For other smaller particle size ranges, limestone consistently showed higher values than basalt and slate, while the particle size distributions for slate and basalt were generally similar. Slate did not exhibit excessive fragmentation issues due to its inherent bedding rock structure. After high-temperature water immersion, high-temperature autoclaving, and high-temperature heating, the particle size distribution of crushed slate aggregate showed little difference from that after conventional crushing test.

Overall, the used slate aggregate exhibited good water resistance and heat resistance. The low crushing value also indicated that its resistance to crushing was comparable to that of traditional basalt and superior to that of limestone aggregate. Furthermore, slate coarse aggregate demonstrated good thermal stability, with no severe strength degradation after high-temperature heating. It had good resistance to crushing during the high-temperature production and construction compaction of asphalt mixtures. All of these results on high crushing resistance and durability could have contributed to the lack of slaty cleavage structure, and the cryptocrystalline matrix prevented these bedding planes from evolving into mechanically weak parting surfaces.

### 3.2. Single-Particle Compression Crushing Strength

The crushing modes of individual aggregate particles were found to be diverse. Taking slate coarse aggregate as an example, typical load-displacement curve results are shown in Figure 6. Overall, the crushing modes of individual particles can be categorized into three types: (1) Single-peak curve: In this crushing mode, the load-displacement curve has only one peak point. The coarse aggregate typically fractures into two pieces with neat fracture surfaces, producing very few fines or small fragments. (2) Double-peak curve: In this crushing mode, the load-displacement curve has two peak points. The main body of the coarse aggregate generally fractures into two pieces, generating a small amount of fines and small fragments. (3) Multi-peak curve: In this crushing mode, the load-displacement curve exhibits multiple peak points. The coarse aggregate usually fractures into multiple fragments, producing a large amount of fines and small fragments.

The statistical results of single-particle crushing modes for a total of 600 aggregate particles from three rock types including limestone, basalt, and slate, are presented in Table 4. For limestone coarse aggregate, regardless of changes in particle size or the number of loading contact points, its load-displacement curves were predominantly characterized by the multi-peak crushing mode, accounting for nearly 50%. The proportions of the other two modes were both close to 26–28%.

For basalt coarse aggregate, during the single-particle two-point compression crushing test, the load-displacement curves were mainly characterized by the double-peak crushing mode, accounting for 40%. The other two modes each accounted for approximately 30%, and particle size had no significant effect on the crushing mode. During the single-particle four-point compression crushing test, the load-displacement curves were dominated by the multi-peak crushing mode. In the 4.75–9.5 mm range, it accounted for 40%, with the other two modes each at 30%. In the 9.5–13.2 mm range, the proportion of the multi-peak crushing mode slightly increased to 44%, while the other two modes each accounted for 28%.

For slate coarse aggregate, during the single-particle two-point crushing test, the load-displacement curves were primarily characterized by the double-peak crushing mode, accounting for about 45%, with the other two modes each around 28%. During the single-particle four-point compression crushing test, the double-peak and single-peak crushing modes were dominant, each accounting for about 40%, while the multi-peak crushing mode accounted for only 16% in both cases.

The presence of weak points on the surface of coarse aggregate particles can lead to poor contact, causing grinding or local fractures that occur before the main failure under load, thereby generating more fines and fragments.

To describe the distribution patterns of compressive crushing strength for the three types of aggregates, the Weibull theory was employed for analysis. Based on the Weibull distribution function, the survival probability of crushable particles is related to their crushing strength, which can be characterized by Equation (4) [17]:

(4) $$P_{s}=\exp\left[-{\left(\frac{\sigma_{f}}{\sigma_{f0}}\right)}^{m}\right]$$

where *P_s_* is the survival probability of the particle, which can be calculated using Equation (5). *σ*_f0_ is the scale parameter, corresponding to the strength at a survival probability of 37%, serving as an important location parameter of the distribution for comparing strength levels of different materials. m is the Weibull modulus (shape parameter). It quantifies the degree of dispersion or reliability of the strength data. A higher m value indicates a more homogeneous material with less scattered strength properties (higher reliability), whereas a lower m value suggests greater inherent variability and defect distribution within the material, leading to less predictable strength.

(5) $$P_{s}=\frac{i}{n+1}$$

where *n* is the total number of test particles. Following conventions in particle crushing strength studies, 50 particles strike a good balance between statistical reliability and experimental effort, sufficient for Weibull distribution fitting. i is the rank of a particle’s crushing strength in the sequence after arranging all particles’ crushing strength data in descending order.

**Figure 7:**
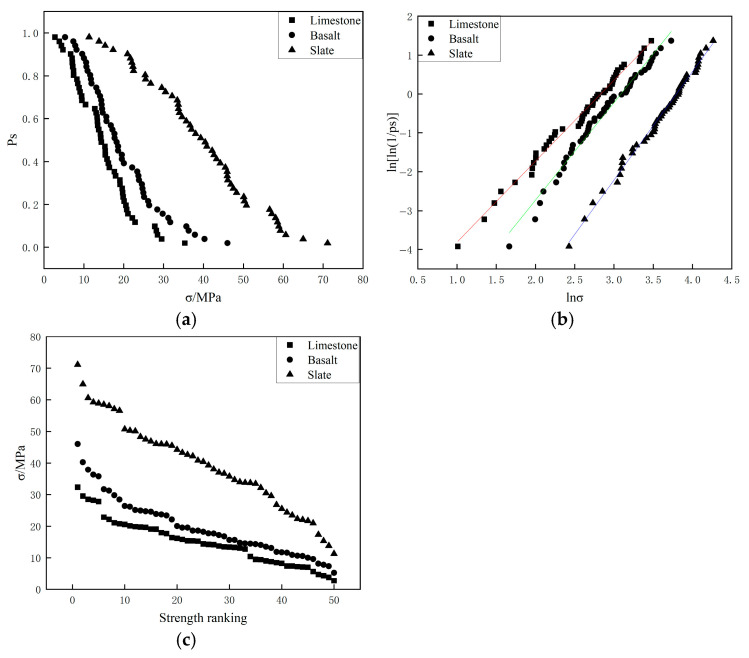
Two-point crushing test results of 4.75–9.5 mm particles for different aggregates ((**a**): Comparison of survival probabilities; (**b**) Weibull distribution of particle strength; (**c**) Single particle strength.).

**Figure 8:**
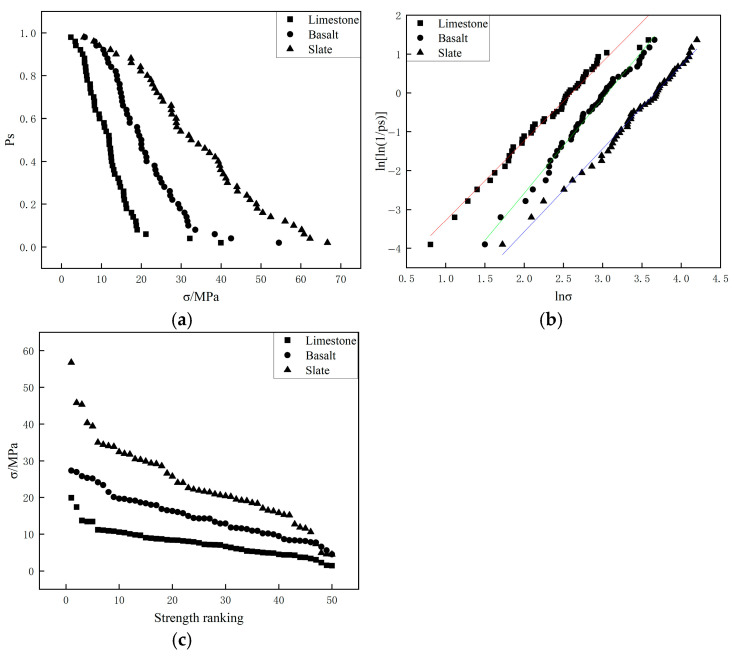
Two-point crushing test results of 9.5–13.2 mm particles for different aggregates ((**a**): Comparison of survival probabilities; (**b**) Weibull distribution of particle strength; (**c**) Single particle strength.).

Figure 7 and Figure 8 present the crushing strength test results for three types of aggregates with different particle sizes (4.75–9.5 mm and 9.5–13.2 mm) from the two-point crushing tests. In particular, the straight lines in Figure 7b and Figure 8b represent the linear fits to the data points, and their slopes correspond to the Weibull modulus. The results showed that the ranking of crushing strength among the three aggregates was consistent with that of the crushing values: slate exhibited the highest crushing strength, followed by basalt, with limestone showing the lowest. In terms of particle survival probability (*P_s_*), slate demonstrated the greatest variability in crushing strength, while basalt and limestone exhibited relatively smaller variations. The Weibull modulus and the characteristic crushing strength corresponding to a 37% survival probability for the different aggregates are listed in Table 5. The fitted correlation coefficients (R^2^) in Table 5 were all greater than 0.98, indicating that the crushing strengths of particles for all three aggregate types followed a Weibull distribution. Overall, the characteristic crushing strength of slate (ranging from 39.77 MPa to 44.42 MPa) was higher than that of basalt (ranging from 20.43 MPa to 21.95 MPa) and limestone (ranging from 12.94 MPa to 17.00 MPa). As the particle size increased, the characteristic crushing strength of all three aggregates decreased. Additionally, the Weibull modulus (ranging from 2.04 to 2.79MPa) also decreased with increasing particle size, which can be attributed to the increase in internal defects within the aggregates as particle size grows, leading to a reduction in crushing strength and an increase in variability.

In terms of particle strength, Ref. [24] conducted single-particle two-point crushing tests on slate particles with different shapes and sizes. The characteristic strength *σ*_f0_ for regularly shaped slate particles ranged from 11.70 to 34.05 MPa, and the Weibull modulus m ranged from 2.026 to 2.697. A similar trend of decreasing characteristic strength and Weibull modulus with increasing particle size was also observed. By comparison, the slate aggregate used in the present study demonstrated a high strength while exhibiting a comparable level of Weibull modulus.

**Figure 9:**
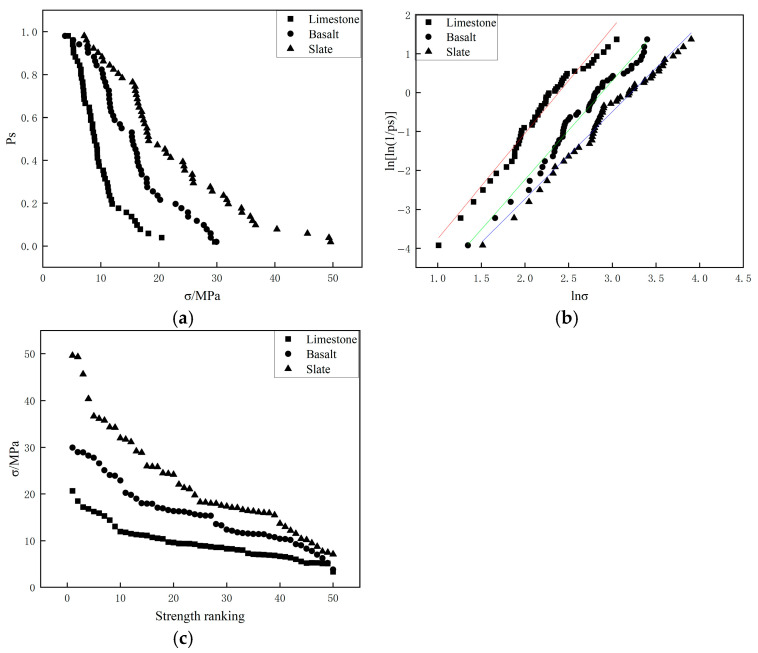
Four-point crushing test results of 4.75–9.5 mm particles for different aggregates ((**a**): Comparison of survival probabilities; (**b**) Weibull distribution of particle strength; (**c**) Single particle strength.).

**Figure 10:**
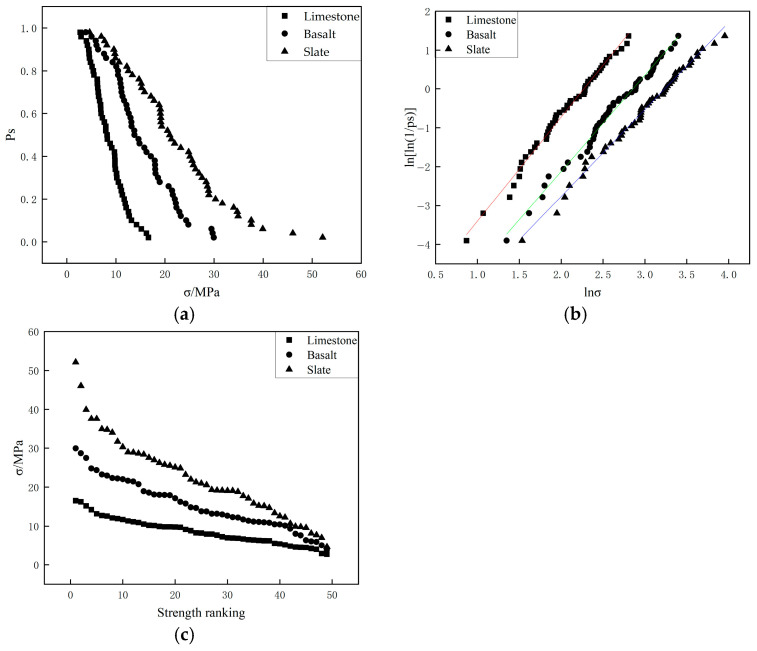
Four-point crushing test results of 9.5–13.2 mm particles for different aggregates ((**a**): Comparison of survival probabilities; (**b**) Weibull distribution of particle strength; (**c**) Single particle strength.).

Figure 9 and Figure 10 present the results of four-point crushing tests for three types of aggregates with different particle sizes (4.75–9.5 mm and 9.5–13.2 mm), respectively. The results of the four-point crushing tests were similar to those of the two-point crushing tests. In the four-point crushing tests, In particular, the straight lines in Figure 9b and Figure 10b represent the linear fits to the data points, and their slopes correspond to the Weibull modulus. the characteristic crushing strength of slate aggregate was consistently higher than that of basalt and limestone aggregates, indicating that slate aggregate exhibited superior crushing strength compared to basalt and limestone. Table 6 provides the Weibull modulus and the characteristic crushing strength corresponding to a 37% survival probability for the different aggregates. As the aggregate particle size increased from 4.75–9.5 mm to 9.5–13.2 mm, the characteristic compressive strength and Weibull modulus for the three types of aggregates in the four-point crushing tests showed minimal changes, indicating low sensitivity to variations in particle size.

The stress states and failure modes of aggregate particles differed between the two-point and four-point crushing tests. Compared to the two-point crushing test, the characteristic crushing strength in the four-point crushing test showed a significant decrease, with a reduction ranging from 20% to 40%, indicating that the stress conditions in the four-point crushing test were more demanding. Overall, the characteristic crushing strengths and Weibull modulus for the four-point crushing tests across different particle sizes were more similar, tending toward a certain value. This suggested that the four-point crushing test was more suitable for evaluating the crushing strength of aggregates with different particle sizes, as it reduced the influence of particle size. Additionally, whether in the two-point or four-point crushing tests, the Weibull modulus for the crushing strength of particles of different sizes for all three aggregates fail within the range of 2.0–3.0. This indicated a relatively dispersed distribution of aggregate crushing strength. The particle size and the number of contact points had a significant influence on the crushing strength.

A comparison of the Weibull modulus for these three types of aggregate (Table 5 and Table 6) revealed that the m values for slate generally ranged from 2.14 to 2.79 across different particle sizes and loading methods. This range partially overlapped with those of limestone (2.04–2.72) and basalt (2.43–2.53) but exhibited a broader variation. This indicated that the dispersion of single-particle crushing strength for slate was at a moderate level, yet its variability is more sensitive to different conditions, such as changes in particle size. A similar Weibull modulus indicates that the tested slate aggregate had a similar variability in the single-particle strength when compared with limestone and basalt.

### 3.3. Coarse Aggregate Compaction Crushing Test

Table 7 presents the results of the crushing resistance tests for single-sized coarse aggregates (9.5–13.2 mm) during compaction. After 75 blows on one side using the Marshall compactor, significant crushing was observed for all aggregate types. For instance, the particle size distribution of limestone aggregates in the 9.5–13.2 mm range was 65.8%, indicating that nearly 34% of the 9.5–13.2 mm aggregate particles were crushed into smaller aggregates. In contrast, the distributions for basalt and slate aggregates in the same size range were 80.6% and 81.0%, respectively, indicating that approximately 20% of the 9.5–13.2 mm aggregate particles were crushed into smaller aggregates. After 75 Marshall compaction blows, limestone aggregates exhibited higher proportions in all smaller particle size ranges compared to basalt and slate aggregates, while the particle size distributions of slate aggregates closely resembled those of basalt.

Compared to the Marshall compaction method, gyratory compaction is another common laboratory compaction method for asphalt mixtures. The gyratory compaction pressure is relatively lower than the Marshall compaction energy, which is reflected in its reduced crushing effect on aggregates. After 205 cycles of gyratory compaction, the particle size distributions of limestone, basalt, and slate aggregates in the 9.5–13.2 mm range were 89.3%, 96.4%, and 94.4%, respectively. This indicated that only about 5% to 10% of the 9.5–13.2 mm aggregate particles were crushed into smaller aggregates, with relatively minimal proportions in other smaller size ranges. These results suggested that the shearing action of gyratory compaction had a less destructive effect on aggregates, primarily causing wear and shearing of aggregate edges and corners. In contrast, Marshall compaction exerted a greater crushing effect, leading to a higher proportion of aggregates in the 4.75–9.5 mm range.

Overall, whether subjected to Marshall compaction or gyratory compaction, slate aggregates demonstrated excellent resistance to crushing. Their particle size distributions across different ranges closely resembled those of basalt, with no evidence of excessive damage due to the inherent bedding structure of slate.

Table 8 presents the results of the crushing resistance tests for mixed coarse aggregates under Marshall compaction and gyratory compaction. The mixed coarse aggregates were composed of three coarse aggregate sizes including 9.5–16 mm, 4.75–9.5 mm, and 2.36–4.75 mm, blended in specific proportions to simulate the skeletal structure formed by all coarse aggregates in asphalt mixtures. After 75 blows on one side using Marshall compaction or 205 cycles of gyratory compaction, the skeletal structures formed by the different coarse aggregates exhibited significant damage, leading to notable changes in their gradation and individual sieve residues. Before testing, the passing percentages of the different mixed aggregates through the 13.2 mm, 9.5 mm, 4.75 mm, and 2.36 mm sieves were 91.7%, 63.9%, 20.8%, and 0%, respectively. After testing, the passing percentages through these sieves increased significantly, with the effect of 75 blows on one side using Marshall compaction being noticeably greater than that of 205 cycles of gyratory compaction.

After 75 blows using Marshall compaction, the limestone mixed aggregates experienced a higher degree of crushing compared to the basalt and slate mixed aggregates, while the passing percentages of the slate mixed aggregates across different sieve sizes closely resembled those of the basalt mixed aggregates. After 205 cycles of gyratory compaction, the differences in passing percentages among the limestone, basalt, and slate mixed aggregates across various sieve sizes were relatively small. This further indicated that the shearing action of gyratory compaction had a less destructive effect on aggregates, whereas Marshall compaction was more demanding and better distinguished the crushing resistance of different aggregates.

**Figure 11:**
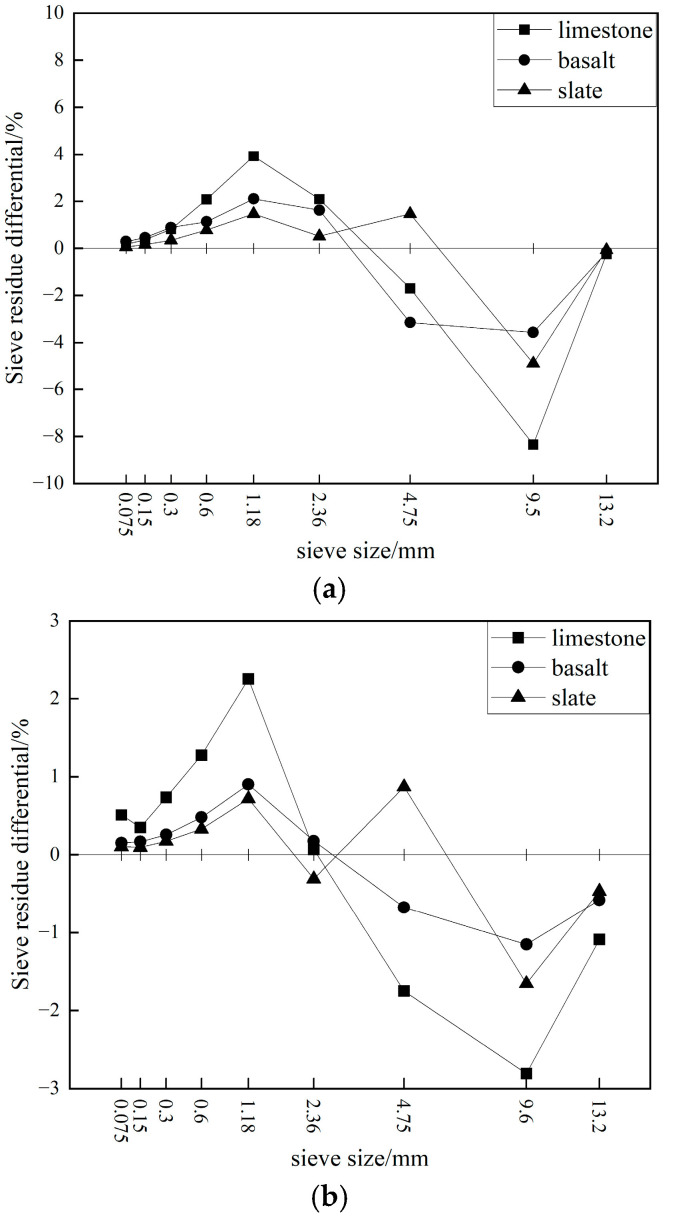
Difference in sieve residue fractions of mixed aggregates after crushing: ((**a**): Marshall compaction; (**b**): gyratory compaction.).

Figure 11 presents the comparative analysis of the differences in individual sieve residues after the crushing of mixed aggregates. It was evident that the skeletal structures composed of different coarse aggregates experienced the most significant crushing in the 9.5–13.2 mm size fraction. For example, after Marshall compaction, the individual sieve residues of the limestone, basalt, and slate mixed aggregates decreased by 8.4%, 3.2%, and 4.9%, respectively. Subsequently, the limestone and basalt mixed aggregates also underwent considerable crushing in the 4.75–9.5 mm fraction, resulting in reductions of 1.7% and 3.6% in their individual sieve residues, respectively. In contrast, the slate mixed aggregates did not experience excessive crushing in the 4.75–9.5 mm fraction; instead, their individual sieve residue increased by 1.4%. Overall, the Marshall compaction process produced the highest amount of fine aggregates in the 1.18–2.36 mm range. For instance, the individual sieve residues of the limestone, basalt, and slate mixed aggregates increased by 3.9%, 2.1%, and 1.5%, respectively.

Compared to Marshall compaction, gyratory compaction had a similar effect on the limestone, basalt, and slate mixed aggregates, with the most significant crushing also occurring in the 9.5–13.2 mm coarse aggregate skeletal structure, and the highest production of fine aggregates in the 1.18–2.36 mm range. However, the magnitude of these changes was relatively smaller. For example, in the 9.5–13.2 mm range, the individual sieve residues of the limestone, basalt, and slate mixed aggregates decreased by 2.8%, 1.2%, and 1.7%, respectively, while in the 1.18–2.36 mm range, they increased by 2.3%, 0.9%, and 0.8%, respectively. This indicated that the damage to the skeletal structure was concentrated in the 9.5–13.2 mm aggregates.

Compared to limestone and basalt, the slate mixed aggregates showed a positive difference in individual sieve residue at the 4.75 mm sieve, indicating that the damage to the skeletal structure of slate coarse aggregates was concentrated only in the 9.5–13.2 mm fraction, demonstrating better skeletal resistance to crushing.

A comparison with the results of the earlier Marshall and gyratory compaction tests on single-sized 9.5–13.2 mm aggregates showed that, for single-sized aggregates, approximately 20% to 34% were crushed after Marshall compaction, while about 5% to 10% were crushed after gyratory compaction. For mixed aggregates, the proportion of crushed aggregates can be characterized by the sum of the differences in individual sieve residues for the three aggregate sizes (9.5–16 mm, 4.75–9.5 mm, and 2.36–4.75 mm). This yielded approximate crushing proportions of 3.0% to 8.0% after Marshall compaction and 1.2% to 4.5% after gyratory compaction. These results indicated that Marshall compaction caused a greater degree of crushing than gyratory compaction, and the skeletal structure formed by mixed aggregates exhibited better resistance to crushing than that of single-sized aggregates. Since Marshall compactors are widely used in laboratories, while gyratory compactors are primarily employed for research purposes, the Marshall compaction test on single-sized aggregates can serve as a rapid and effective method for evaluating the crushing resistance of coarse aggregates.

In the compaction tests on coarse aggregates (both single-size and blended gradation), although minor deviations existed among different replicate tests, the range of variation was normal and controllable, and the exhibited trends in gradation evolution were highly consistent. Comparisons with reference aggregates clearly show that under both Marshall impact and gyratory shear compaction, the degree of breakage and gradation curves of slate aggregate were very close to those of basalt aggregate. These results demonstrate, from the scale of a pure aggregate skeleton, that slate aggregate can form a stable skeletal structure under compaction energy simulating construction. Its relict bedding structure did not lead to abnormal or excessive breakage of the skeleton, indicating excellent resistance to compaction-induced crushing.

### 3.4. Asphalt Mixture Compaction Crushing Test

Table 9 presents the results of the crushing resistance tests for different asphalt mixtures under Marshall compaction and gyratory compaction. In these tests, all asphalt mixtures used the same gradation to ensure a consistent aggregate skeletal structure. After 75 blows on each side using Marshall compaction or 205 cycles of gyratory compaction, significant changes were observed in the gradation and individual sieve residues of the different asphalt mixtures. Before testing, the passing percentages of the different asphalt mixtures through the key sieve sizes of 9.5 mm, 4.75 mm, 2.36 mm, and 0.075 mm were 74.0%, 43.0%, 26.0%, and 5.8%, respectively. After testing, the passing percentages through these key sieve sizes increased noticeably, with the effect of 75 blows on each side using Marshall compaction being significantly greater than that of 205 cycles of gyratory compaction.

While minor deviations exist among the replicate test data in Table 9, the overall trends indicated that the gradation changes of slate asphalt mixture after Marshall and gyratory compaction closely overlapped with the distribution range of basalt asphalt mixture. Their variation was distinctly different from the change curve of limestone asphalt mixture. This clear data distribution and evolution trend indicated that the compaction-induced breakage behavior of slate aggregate in asphalt mixtures is fairly consistent with that of basalt aggregate.

After 75 blows on each side using Marshall compaction, the limestone asphalt mixture experienced slightly more crushing compared to the basalt and slate asphalt mixtures, with increases in passing percentages across different sieve sizes ranging from 2% to 4%. In contrast, after 205 cycles of gyratory compaction, the differences in passing percentages among the limestone, basalt, and slate asphalt mixtures across various sieve sizes were minimal, all within 2%.

**Figure 12:**
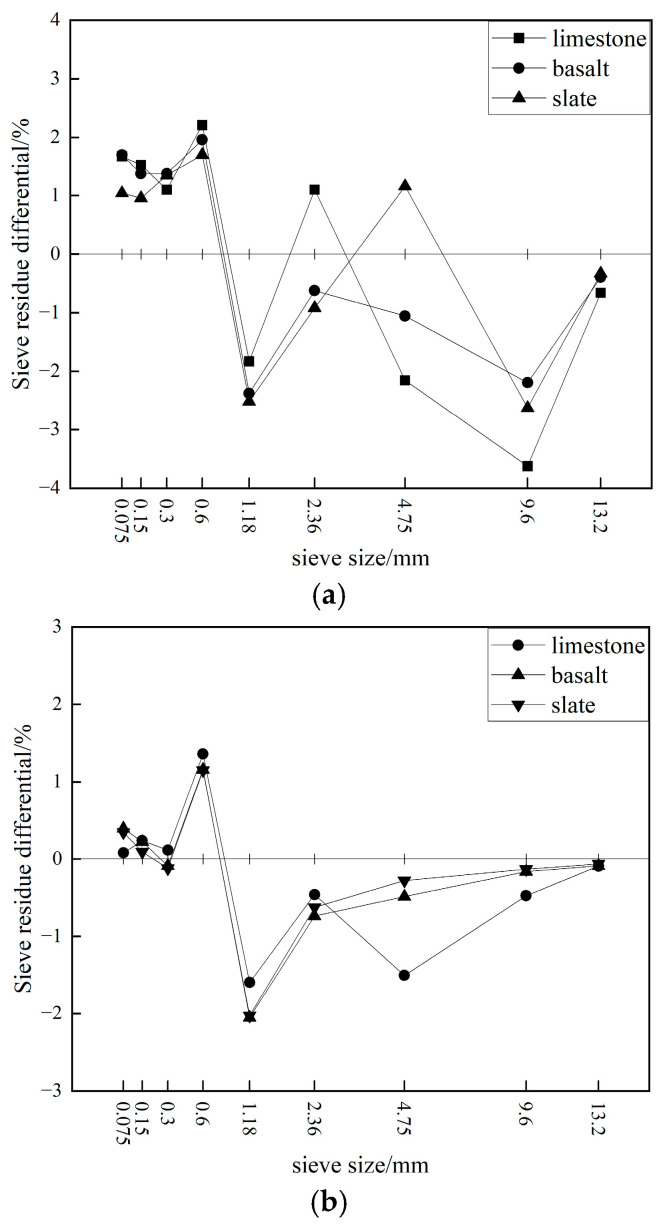
Difference in sieve residue fractions of asphalt mixture after crushing: (**a**) Marshall compaction; (**b**) gyratory compaction.

Figure 12 presents the comparative analysis of the differences in individual sieve residues among the different asphalt mixtures; it was evident that the effects of Marshall compaction and gyratory compaction on asphalt mixtures differed significantly. For example, Marshall compaction primarily caused the most significant crushing in the 9.5–13.2 mm aggregates, followed by the 1.18–2.36 mm aggregates. In contrast, gyratory compaction resulted in the most significant crushing in the 1.18–4.75 mm aggregates. In terms of the increase in individual sieve residues, Marshall compaction mainly led to an increase in fine materials within a broader range of 0.075–0.6 mm, while gyratory compaction primarily caused an increase in materials smaller than 0.075 mm (filler) and fine materials in the 0.6–1.18 mm range. This indicated that, under standard laboratory compaction conditions for asphalt mixtures, the destructive effects of Marshall compaction and gyratory compaction on aggregates were entirely different. The former primarily involved the crushing and refinement of coarse aggregates due to impact, while the latter mainly involved the grinding and shearing of 1.18–4.75 mm aggregates under rotational shear forces.

Based on a comparative analysis with the results of the crushing resistance tests on mixed aggregates under gyratory compaction, the findings described above were related to the fact that the asphalt mixture used limestone for the 0–2.36 mm fine aggregates, while basalt and slate, which are harder, were used for the coarse aggregates. The limestone fine aggregates filled the gaps in the skeletal structure formed by the basalt and slate coarse aggregates, making them more prone to breakage under compression and grinding. Overall, after adding asphalt binder, mineral filler, and fine aggregates, the degree of crushing of each type of aggregate in the asphalt mixture was significantly lower than that observed in the aforementioned mixed aggregates.

Similarly, the proportion of crushed coarse aggregate skeletal structure in the asphalt mixture can also be characterized by the sum of the residual differences for the three aggregate sizes: 9.5–16 mm, 4.75–9.5 mm, and 2.36–4.75 mm. Based on this, the proportion of aggregates crushed after Marshall compaction was approximately 2.5% to 4.7%, whereas after gyratory compaction was about 1.0% to 2.5%. These results further indicated that the degree of crushing caused by Marshall compaction was much greater than that caused by gyratory compaction, which can easily lead to internal structural damage in the specimen and affect its mechanical properties.

Whether under Marshall compaction or gyratory compaction, the resistance to crushing progressively improved from the skeletal structure formed by single-sized aggregates to that of mixed aggregates and further to the skeletal structure of asphalt mixtures. This also underscored that when designing asphalt mixtures with a high-skeletal structure, high-quality, crush-resistant aggregates should be selected, while excessive compaction that could cause aggregate breakage should be avoided. Slate asphalt mixtures demonstrated excellent resistance to crushing, comparable to basalt asphalt mixtures, which further confirms that the metamorphic bedding structure of slate did not lead to excessive aggregate crushing in its asphalt mixtures.

In the compaction tests on all asphalt mixtures, all results point to a clear trend that the degree of compaction-induced breakage in slate asphalt mixture was at the same level as that of basalt asphalt mixture and significantly lower than that of limestone asphalt mixture. This result further confirms, from the engineering scale of a complete asphalt mixture, that the internal structure of slate aggregate skeleton can withstand complex multi-directional stresses during actual mixture formation and compaction. No excessive breakage due to potential weak planes in slate aggregate occurred. It should be noted that an asphalt mixture is a multi-particle system where inter-particle interlocking is more sensitive to weaker particles, which are prone to be crushing under compaction. The compaction tests done on mixed aggregates and asphalt mixtures in this study showed that the tested slate aggregate did not exhibit excessive crushing in the stone skeleton, confirming the reliability of its overall performance at the engineering scale.

## 4. Conclusions

A series of crushing resistance tests was conducted on slate, limestone, and basalt aggregates. The crushing modes, crushing strength, and gradation changes of slate aggregates and its asphalt mixture was analyzed, and the main conclusions are as follows:

(1) The tested slate coarse aggregate exhibits a low crushing value, with its resistance to crushing comparable to that of traditional basalt and superior to limestone aggregate. The slate aggregate demonstrates good water resistance and thermal stability, meeting the requirements for resistance to crushing during high-temperature production and construction compaction of asphalt mixtures, as well as the demands for weathering resistance during service.
(2) For the tested slate coarse aggregate, the load-displacement curves from single-particle two-point compression crushing tests are primarily characterized by a double-peak crushing mode. In single-particle four-point compression crushing tests, the dominant modes are double-peak and single-peak crushing. The single-particle compressive crushing strength of slate coarse aggregate is greater than that of limestone and basalt aggregates, and the compressive crushing strength of individual particles follows the Weibull distribution pattern. Aggregate particle size and the number of force contact points significantly influence its characteristic crushing strength and Weibull modulus. The four-point crushing test is more suitable than the two-point crushing test for evaluating the crushing strength of aggregates with different particle sizes, as it reduces the influence of particle size.
(3) Marshall compaction causes a greater degree of crushing in different aggregates compared to gyratory compaction. Additionally, single-sized aggregates experience 2–3 times higher crushing levels than mixed aggregates. Therefore, the Marshall compaction test on single-sized aggregates can be used to rapidly evaluate the crushing resistance of aggregates from different rock types.
(4) Both the tested single-sized and mixed slate aggregates exhibit lower proportions of aggregate breakage during Marshall compaction and gyratory compaction processes compared to basalt and limestone aggregates. Asphalt mixtures prepared using slate coarse aggregate also demonstrate excellent resistance to crushing compared to basalt asphalt mixtures. This further confirms that the inherent bedding structure of the tested slate does not lead to excessive aggregate crushing and damage in its asphalt mixtures.

This study only focused on one type of slate, limestone, and basalt, and only one type of asphalt mixture was considered. The obtained findings were limited to the tested slate aggregates from a single quarry and thus necessary performance verification should be conducted on slate aggregates from other sources before practical engineering applications. In order to further validate the results of this study, a test section of 300 m of asphalt concrete containing the slate coarse aggregates obtained from the De’an Quarry in Qinzhou was constructed in the Qinzhoubei Freeway, April, 2025. The tracking observation results of the test section will further verify the long-term service performance of asphalt concrete with slate aggregates.

## Figures and Tables

**Table 1:** The mineral gradation of AC-13 asphalt mixture.

Type of Gradation	Passing Percentage at Different Sieve Sizes									
	16	13.2	9.5	4.75	2.36	1.18	0.6	0.3	0.15	0.075
Upper limit	100	100	75	39	30	22	18	14	11	8
Lower limit	100	80	62	25	18	14	8	6	5	5
Designed gradation	100	94	74	43	26	17.9	11.7	8.0	6.4	5.8

**Table 2:** Crushing value test results of different coarse aggregates.

Aggregate	Limestone	Basalt	Slate	Slate (Soaked in Water at 60 °C for 21 Days)	Slate (Autoclaved at High Temperature)	Slate (Heated at 220 °C for 4 h)
Crushing value (%)	19.8	11.4	9.2	10.2	10.6	10.6

**Table 3:** Particle size distribution of crushed aggregates after the crushing value test.

Aggregate Particle Size	9.5–13.2	4.75–9.5	2.36–4.75	1.18–2.36	0.6–1.18	0–0.6
Limestone	22.9	40.0	17.3	7.5	5.6	6.7
Basalt	46.4	33.2	9.0	3.5	3.0	4.9
Slate	44.8	35.0	11.1	3.4	2.7	3.1
Slate (soaked in water at 60 °C for 21 days)	41.1	36.8	11.9	3.8	3.0	3.4
Slate (autoclaved at high temperature)	41.4	36.9	11.1	4.2	3.1	3.3
Slate (heated at 220 °C for 4 h)	39.3	38.2	11.9	4.2	3.1	3.3

**Table 4:** Breakdown of fracture modes for single-particle compression crushing tests.

Aggregate	Loading Method	Particle Size Distribution	AR	Breakdown of Fracture Modes (%)		
				Single-Peak	Double-Peak	Multi-Peak
Limestone	Two-point crushing	4.75–9.5 mm	1.0–1.2	26	28	46
		9.5–13.2 mm	1.0–1.2	28	26	46
	Four-point crushing	4.75–9.5 mm	1.0–1.2	26	28	46
		9.5–13.2 mm	1.0–1.2	26	26	48
Basalt	Two-point crushing	4.75–9.5 mm	1.0–1.2	32	40	28
		9.5–13.2 mm	1.0–1.2	28	40	32
	Four-point crushing	4.75–9.5 mm	1.0–1.2	30	30	40
		9.5–13.2 mm	1.0–1.2	28	28	44
Slate	Two-point crushing	4.75–9.5 mm	1.0–1.2	28	46	26
		9.5–13.2 mm	1.0–1.2	28	44	28
	Four-point crushing	4.75–9.5 mm	1.0–1.2	40	44	16
		9.5–13.2 mm	1.0–1.2	44	40	16

**Table 5:** Statistical results of two-point crushing tests for different aggregates.

Aggregate	Aggregate Particle Size	Characteristic Compressive Strength/(MPa)	m	R^2^
Limestone	4.75–9.5 mm	17.00	2.08	0.989
	9.5–13.2 mm	12.94	2.04	0.980
Basalt	4.75–9.5 mm	21.95	2.50	0.982
	9.5–13.2 mm	20.43	2.43	0.980
Slate	4.75–9.5 mm	44.42	2.79	0.993
	9.5–13.2 mm	39.77	2.14	0.990

**Table 6:** Statistical results of four-point crushing tests for different aggregates.

Aggregate	Aggregate Particle Size	Characteristic Compressive Strength/(MPa)	m	R^2^
Limestone	4.75–9.5 mm	9.71	2.72	0.975
	9.5–13.2 mm	9.81	2.67	0.989
Basalt	4.75–9.5 mm	17.70	2.53	0.980
	9.5–13.2 mm	17.97	2.48	0.989
Slate	4.75–9.5 mm	24.33	2.24	0.983
	9.5–13.2 mm	25.65	2.23	0.993

**Table 7:** Crushing resistance test results of single-sized 9.5–13.2 mm aggregates under Marshall and gyratory compaction.

Aggregate	Compaction Method	9.5–13.2	4.75–9.5	2.36–4.75	1.18–2.36	0.6–1.18	0–0.6
Limestone	Marshall compaction	65.8	24.8	4.9	1.7	1.2	1.6
	Gyratory compaction	89.3	7.0	1.4	0.6	0.6	1.1
Basalt	Marshall compaction	80.6	14.3	2.4	0.8	0.6	1.3
	Gyratory compaction	96.4	2.5	0.5	0.1	0.1	0.5
Slate	Marshall compaction	81.0	15.4	2.1	0.7	0.5	0.5
	Gyratory compaction	94.4	4.4	0.4	0.2	0.2	0.4

**Table 8:** Crushing resistance test results of mixed aggregates under Marshall and gyratory compaction.

Sieve Size/mm	Limestone		Basalt		Slate		Designed Gradation
	Marshall Compaction	Gyratory Compaction	Marshall Compaction	Gyratory Compaction	Marshall Compaction	Gyratory Compaction	
13.2	91.9	92.8	91.8	92.2	91.7	92.1	91.7
9.5	72.5	67.8	67.2	65.6	68.8	66.0	63.9
4.75	31.1	26.5	27.7	23.2	24.3	22.1	20.8
2.36	8.2	5.6	5.2	2.2	3.0	1.6	0.0
1.18	4.3	3.3	3.1	1.3	1.5	0.8	0.0
0.6	2.2	2.0	2.0	0.9	0.7	0.5	0.0
0.3	1.4	1.3	1.1	0.6	0.4	0.3	0.0

**Table 9:** Crushing resistance test results of asphalt mixture under Marshall and gyratory compaction.

Sieve Size/mm	Limestone		Basalt		Slate		Designed Gradation
	Marshall Compaction	Gyratory Compaction	Marshall Compaction	Gyratory Compaction	Marshall Compaction	Gyratory Compaction	
13.2	94.7	94.1	94.4	94.1	94.3	94.1	94.0
9.5	78.3	74.6	76.6	74.2	77.0	74.2	74.0
4.75	49.4	45.0	46.6	43.7	44.8	43.4	43.0
2.36	31.4	28.6	30.3	27.5	28.8	27.1	26.0
1.18	25.0	22.0	24.5	21.4	23.1	21.0	17.9
0.6	16.7	14.5	16.4	14.1	15.3	13.7	11.7
0.3	11.8	10.6	11.3	10.4	10.1	10.1	8.0
0.15	8.7	8.8	8.3	8.6	7.6	8.4	6.4
0.075	6.4	8.1	6.0	7.6	6.0	7.5	5.8

## Data Availability

The original contributions presented in this study are included in the article. Further inquiries can be directed to the corresponding author.

## References

[1] Ikari M.J., Niemeijer A.R., Marone C. (2015). Experimental investigation of incipient shear failure in foliated rock. J. Struct. Geol..

[2] Bahaaddini M., Hagan P.C., Mitra R., Khosravi M.H. (2014). Scale effect on the shear behaviour of rock joints based on a numerical study. Eng. Geol..

[3] Wenk H.-R., Yu R., Cárdenes V., López-Mungira A., Briers N. (2020). Fabric and anisotropy of slates: From classical studies to new results. J. Struct. Geol..

[4] Oti E.J. (2010). Engineering properties of concrete made with slate waste. Proc. Inst. Civ. Eng.-Constr. Mater..

[5] Chen Y., Wei K., Liu W., Yang C.H., Zhou J.W. (2016). Experimental characterization and micromechanical modelling of anisotropic slates. Rock Mech. Rock Eng..

[6] Ding C.-D., Zhang Y., Hu D.-W., Zhou H., Shao J.-F. (2020). Foliation effects on mechanical and failure characteristics of slate in 3D space under Brazilian test conditions. Rock Mech. Rock Eng..

[7] Weng M.-C., Lin S.-S., Lee C.-S., Jeng F.-S. (2024). An anisotropic thermal–mechanical coupling failure criterion for slate. Rock Mech. Rock Eng..

[8] Weng M.-C., Li H.-H., Fu Y.-Y., Fang C.-H., Chen H.-R., Chang C.-Y. (2022). A failure criterion for foliation and its application for strength estimation of foliated metamorphic rock. Int. J. Rock Mech. Min. Sci..

[9] Weng M.-C., Chang C.-Y., Jeng F.-S., Li H.-H. (2020). Evaluating the stability of anti-dip slate slope using an innovative failure criterion for foliation. Eng. Geol..

[10] Wu H., Ma T., Li Y., Yang T., Zhang G. (2023). Numerical and experimental investigation of the anisotropic tensile behavior of layered rocks in 3D space under Brazilian test conditions. Int. J. Rock Mech. Min. Sci..

[11] Debecker B., Vervoort A. (2009). Experimental observation of fracture patterns in layered slate. Int. J. Fract..

[12] Hong R.-C., Yu R., He T., He M.-C. (2020). Failure and mechanical behavior of transversely isotropic rock under compression-shear tests: Laboratory testing and numerical simulation. Eng. Fract. Mech..

[13] Xu Y.-P., Cheng Q.-N., Huang X.-C., Yang G.-S., Zhou L.-F. (2020). An experimental study of microstructure and uniaxial compression test of carbonaceous slate in a deep buried tunnel. Hydrogeol. Eng. Geol..

[14] Jiang H., Jiang A., Zheng F. (2024). Creep mechanical behavior and damage model of layered slate under combined thermal-hydraulic-mechanical action. Case Stud. Therm. Eng..

[15] Yang C.-H., Mao H.-J., Wang H.-L., Li S.-C. (2006). Study on variation of microstructure and mechanical properties of water-weakening slates. Rock Soil Mech..

[16] Li E., Feng J., Zhang L., Chen Q., Wang P. (2021). Brazilian tests on layered carbonaceous slates under water-rock interaction and weathering. Chin. J. Geotech. Eng..

[17] Li G., Tang C. (2015). A statistical meso-damage mechanical method for modeling trans-scale progressive failure process of rock. Int. J. Rock Mech. Min. Sci..

[18] Xiang H., Zhang W., Liu P., He Z. (2020). Fatigue–Healing Performance Evaluation of Asphalt Mixture Using Four-Point Bending Test. Mater. Struct..

[19] Zheng P., Tan X., Du Z., Chen L., Liu H., Wang D. (2025). Microscopic damage and deterioration of carbonaceous slate in cold region subjected to freeze-thaw cycles. J. Rock Mech. Geotech. Eng..

[20] Zheng P., Tan X., Xie Y., Wang F., Liu H. (2025). Study on the tensile strength and fracture characteristics of interlayer mineral grain interfaces in slate exposed to water–rock interaction. Rock Mech. Rock Eng..

[21] Liu H., Qin H., Gao Y., Zhou Y. (2005). Experimental study on particle breakage of rockfill and coarse aggregates. Rock Soil Mech..

[22] Jiang S., Yan H., Mo L. (2022). Crushing characteristics of coarse aggregates for asphalt mixtures under simulated laboratory compaction loads and repeated traffic loads. Materials.

[23] Shen C., Liu S., Wang L., Li X., Xu W. (2019). Micromechanical modeling of particle breakage of granular materials in the framework of thermomechanics. Acta Geotech..

[24] Gao Y., Mao H. (2025). Impacts of slow-rate cyclic loading on the crushing of single rockfill particles. Rock Mech. Rock Eng..

[25] (2025). Test Methods of Aggregate for Highway Engineering.

[26] Jaeger J.C. (1967). Failure of rocks under tensile conditions. Int. J. Rock Mech. Min. Sci. Geomech. Abstr..

[27] Afshar T., Disfani M.M., Arulrajah A., Narsilio G.A. (2017). Impact of particle shape on breakage of recycled construction and demolition aggregates. Powder Technol..

[28] Todisco M.C., Wang W., Coop M.R., Senetakis K. (2017). Multiple contact compression tests on sand particles. Soils Found..

